# Impact of relative timing of low salinity and polymer flooding on mechanisms by which oil recovery is improved

**DOI:** 10.1038/s41598-025-12863-1

**Published:** 2025-07-25

**Authors:** Ebuka David, Steven R. McDougall, Eric J. Mackay

**Affiliations:** Institute of GeoEnergy Engineering, Heriot-Watt University, Edinburgh, EH14 4AS Scotland

**Keywords:** Enhanced oil recovery (EOR), Polymer flooding, Low salinity (LS) brine, Injection timing, Injection sequence, Synergy, Fossil fuels, Fuel cells

## Abstract

A combination of enhanced oil recovery (EOR) methods, specifically polymer flooding and low salinity (LS) brine injection, has been shown to improve oil recovery beyond what is achievable with either method used alone. However, the optimal sequence and timing of these methods remain unclear, affecting their efficiency. This study investigates the impact of injection sequences and timing of LS brine and polymer to optimize oil recovery by understanding the underlying mechanisms. Six injection scenarios were tested: (1) injecting high salinity (HS) water followed by LS brine (tertiary injection), (2) injecting HS water to intermediate saturation followed by LS brine, (3) injecting LS brine directly (secondary injection), and in each case, (4) polymer injected simultaneously with LS brine, (5) polymer injected after the LS brine, or (6) polymer injected before the LS brine. The results showed a positive synergy between LS brine and polymer in both secondary and tertiary injections. This synergy is highly sensitive to injection timing, sequence, and rock/fluid properties. The combined effect of LS brine and polymer shifts the flow regime by altering the balance between capillary and viscous forces, maximizing oil recovery when both mechanisms are active. Conversely, the effectiveness declines when one mechanism dominates. Therefore, the timing and order of polymer and LS brine injection significantly influence displacement efficiency and oil recovery, with different injection sequences producing varying outcomes, even with the same EOR techniques.

## Introduction

 Enhanced oil recovery (EOR) techniques play a critical role in maximizing hydrocarbon extraction from reservoirs, particularly in mature fields where conventional production methods have become inefficient^1^. Among the diverse EOR methods developed over the decades, polymer flooding and low salinity water flooding (LSWF) have emerged as two of the most promising water-based techniques due to their relatively lower operational costs and demonstrated recovery potential.

Polymer flooding involves the injection of high-molecular-weight, water-soluble polymers—typically partially hydrolyzed polyacrylamide (HPAM)—to increase the viscosity of the injected water, thereby reducing the mobility ratio between water and oil^2^. This enhances macroscopic sweep efficiency and suppresses viscous fingering, a problem particularly prominent in high-permeability streaks or heterogeneously layered reservoirs. Field-scale implementations of polymer flooding date back several decades, with some of the most well-documented applications being the Oerrel and Hankensbuettel fields in Germany^3^, the Daqing oil field in China^4^, and the Tambaredjo field in Suriname^5^. These projects have demonstrated substantial incremental oil recovery, but also exposed challenges related to polymer retention, shear degradation, and salinity sensitivity, particularly in carbonate and high-temperature reservoirs.

In contrast, low salinity water flooding (LSWF) re-emerged in the mid-1990s as a promising EOR technique following the seminal work of Morrow (1996)^6–8^. Unlike polymer flooding, which enhances mobility control, LSWF primarily acts through wettability alteration, reducing residual oil saturation by shifting the rock from an oil-wet or mixed-wet state toward a more water-wet condition. This transition lowers capillary forces that trap oil and promotes more favorable relative permeability conditions for oil displacement^9^. Various mechanisms have been proposed to explain the effects of low salinity injection, including multicomponent ion exchange, expansion of the electrical double layer, pH increase, and fine migration, although the dominant mechanism remains the subject of ongoing investigation^10^. Despite encouraging laboratory-scale results, field-scale applications of LSWF have been limited and met with mixed success, largely due to uncertainties in salinity thresholds, compatibility with formation water and rock mineralogy, and long-term injectivity performance^11^.

Recognizing the complementary strengths of these two methods—mobility control from polymer and wettability alteration from low salinity brine—there has been increasing research interest in combining them into a hybrid process known as low salinity polymer flooding (LSPF)^12,13^. This concept aims to enhance recovery by integrating the benefits of each individual process, and it has been supported by experimental studies showing that polymers tend to retain higher viscosities and improved propagation behavior in low salinity environments. Laboratory experiments have demonstrated that polymer solutions prepared in low salinity brine exhibit reduced adsorption, higher retention times, and better viscoelastic properties under certain conditions^14–16^. These synergistic effects are especially relevant for heterogeneous sandstone and carbonate reservoirs, where achieving stable displacement fronts and accessing tighter pore spaces remain critical challenges.

The theoretical underpinning of LSPF was further advanced by Khorsandi et al. (2017)^17^, who developed an analytical model to describe the combined effects of wettability alteration and mobility control. However, while their work represented an important step forward, there remains a notable lack of field-level evidence supporting LSPF. To date, only limited pilot studies and coreflood validations have been published, and most research remains confined to the laboratory and simulation scales. This lack of field-scale performance indicators limits the widespread adoption of LSPF, despite its apparent potential. Furthermore, while most studies have focused on brine composition and polymer type, the question of when to inject low salinity brine relative to polymer—i.e., the relative timing and sequence of injection—has received far less attention.

Recent experimental work has begun to highlight the significance of injection timing. Core flooding studies by Yousef et al. (2020)^18^ revealed that injecting low salinity brine prior to high salinity breakthrough resulted in more uniform wettability alteration and greater recovery than injecting it after residual oil saturation was reached. Similarly, Wang et al. (2022)^19^ demonstrated that the combination of low salinity pre-conditioning followed by polymer injection led to higher recovery rates in sandstone cores, attributed to the expansion of accessible pore networks and enhanced mobility control. Visual micromodel experiments by Fadaei et al. (2023)^20^ provided further confirmation that early LS brine injection suppressed viscous fingering, enabling the subsequently injected polymer to form broader and more stable displacement fronts. These findings suggest that the relative order of injection significantly influences the efficiency of the process.

In addition, advanced imaging studies such as those by Zhang et al. (2024)^21^ have shown that pore-scale wettability changes induced by LS brine injection can affect flow paths and displacement fronts well before polymer enters the system. Conversely, injecting polymer prior to LS brine may lead to early path establishment that limits the ability of LS brine to access high capillary entry pressure pores. This asymmetry highlights a critical yet underexplored parameter in LSPF design: the relative timing of each EOR fluid.

Despite the growing evidence pointing to the importance of injection sequence, there is currently no systematic study that directly addresses how the timing of low salinity brine and polymer injection influences oil recovery across different flow regimes and pore structures. Most existing literature evaluates fixed sequences or neglects the pore-scale mechanisms that control displacement efficiency. Consequently, there is a pressing need to evaluate this interaction through a framework that can capture both the fluid dynamics and wettability evolution at the pore scale.

This study addresses this gap by systematically investigating the role of injection timing in low salinity polymer flooding using a pore-scale capillary network model. The objective is to isolate and quantify the effects of sequence timing on oil recovery, displacement stability, and wettability-induced flow regime transitions. The model integrates capillary pressure effects, contact angle variability, and mobility contrasts to simulate realistic displacement scenarios. By comparing different injection sequences across multiple network realizations and analyzing the resulting Regime Index evolution, this work aims to provide mechanistic insights and practical guidance for optimizing LSPF designs in both experimental and field-scale applications.

### Polymer flooding: Pore-Scale mechanisms

Polymer flooding has been extensively studied for its ability to increase oil recovery by improving macroscopic sweep efficiency. However, understanding the microscale phenomena is critical to optimizing this process. The dynamic interaction between capillary and viscous forces is a key determinant of displacement efficiency during water and polymer flooding. Chen et al. (2017)^22^ experimentally visualized the transition from capillary fingering to viscous fingering in rough fractures, showing how flow regime shifts depend on injection rate and fluid viscosity. Their work underscores the importance of capturing these transitions in simulation studies, especially when evaluating timing-sensitive EOR methods that rely on controlled wettability alteration and mobility modification, such as low salinity polymer flooding.

Furthermore, the viscoelastic properties of polymer solutions introduce additional complexity during porous media flow. Rock et al. (2017)^23^ reported the occurrence of elastic instabilities and flow deviations in laboratory polymer floods, highlighting that polymer performance is not only governed by viscosity but also by elastic stresses that emerge under certain flow conditions. These behaviors can influence sweep patterns, pressure gradients, and residual oil mobilization—factors that are highly relevant when evaluating the synergy between low salinity brine and polymer injection at the pore scale.

Clemens et al. (2012)^24^ emphasized the importance of pore-scale modeling in understanding polymer flooding mechanisms. In their micromodel experiments using realistic pore geometries, they found that polymer injection reduced viscous fingering, which occurs when the displacing fluid moves through higher permeability zones, bypassing large portions of the reservoir. By reducing these instabilities, polymer flooding ensures a more uniform sweep of the oil, increasing overall recovery efficiency.

Further, Clemens et al. (2012)^25^ used computational fluid dynamics (CFD) simulations to investigate pore-scale displacements, observing that both secondary and tertiary polymer injections significantly reduced the number and length of water fingers in more permeable pathways. These findings suggest that polymers not only enhance macroscopic efficiency but also exert a strong influence on the microscopic flow patterns within the reservoir^26^. Despite the advancements, pore-scale modeling of polymer flooding remains relatively limited, and many experiments have failed to account for critical phenomena like shear thinning and thickening, which are essential for capturing the full behavior of polymer solutions under varying flow conditions.

### Low-Salinity water flooding: wettability and sweep efficiency

Low-salinity water flooding (LSWF) has gained traction due to its ability to modify the wettability of the reservoir rock, especially in oil-wet formations^27–29^. By reducing the salinity of the injection water, LSWF can induce changes in the electrostatic interactions between the rock surface and the injected brine, leading to a shift from oil-wet to water-wet conditions. Watson et al. (2017)^30,31^ developed a pore network model to study low-salinity brine displacement, highlighting how changes in local wettability can redistribute pore sizes and alter sweep efficiency. Their results suggested that the dynamic modification of contact angles at the pore scale was a critical factor in determining the success of low-salinity floods.

Studies by Amirian & Haghighi (2018)^32^ also demonstrated the pore-scale mechanisms of LSWF using clay-coated glass micromodels. Their work showed that, in water-wet systems, LSWF suppressed snap-off, leading to more stable oil-water interfaces and improved oil displacement. In oil-wet systems, LSWF promoted a shift towards water-wet conditions, primarily driven by double-layer expansion, while fines migration was found to be insignificant. These findings underscore the versatility of low-salinity water flooding in altering wettability, which is crucial for enhancing oil recovery from reservoirs with complex wettability distributions.

#### Pore-Scale digital analysis

Bakke and Øren (1997)^33^ were among the first to establish realistic three-dimensional digital rock models for sandstones, demonstrating how pore geometry and connectivity influence flow pathways and saturation distribution. Such modeling efforts have since laid the groundwork for detailed investigations into the impact of fluid properties and injection strategies on displacement efficiency—particularly relevant for hybrid EOR processes such as low salinity and polymer flooding.

Recent advancements in pore-scale digital rock analysis have provided deeper insights into the mechanisms of polymer and low-salinity water flooding. Yakimchuk et al. (2020)^34^ used digital rock models to analyze relative permeability curves and pore-level displacement efficiencies during polymer flooding. Their findings highlighted the importance of the oil-to-displacing fluid viscosity ratio in determining the dominant recovery mechanism. As this ratio decreased, the process transitioned from viscosity-dominated instability to velocity stripping, demonstrating the dynamic nature of EOR processes at the pore scale.

In a similar vein, Aziz et al. (2019)^35^ used direct numerical simulations to explore the mixing of low- and high-salinity water during polymer flooding. Their results indicated that slow mixing in stagnant regions of the pore network limited the effectiveness of wettability alteration, particularly in tertiary low-salinity flooding. Conversely, flowing regions experienced more rapid wettability changes, improving oil recovery. This research underscores the importance of understanding the fluid dynamics within pore spaces to optimize the combination of polymer and low-salinity water flooding techniques.

### Synergies between polymer and Low-Salinity water flooding

The combination of polymer flooding and LSWF has the potential to significantly enhance oil recovery beyond what either method can achieve individually. Skauge & Shiran (2013)^36^ investigated the synergy between these two processes, finding that polymers were more effective in mobilizing trapped oil in low-salinity environments. They observed that polymer injection at initial water saturation resulted in higher recovery rates compared to injection at residual oil saturation. This is because tertiary low-salinity floods encounter trapped oil that is difficult to mobilize, whereas secondary low-salinity floods face a continuous oil phase, preventing oil trapping in the first place.

Additionally, the combination of low-salinity brine and polymer can improve mobility control by stabilizing the displacement front and reducing viscous fingering. This synergy leads to a better mobility ratio and improved sweep efficiency, allowing the injected water to reach previously inaccessible pore volumes. Mohammadi & Jerauld (2012)^15^ further highlighted the economic benefits of this combination, reporting that the amount of polymer required for flooding could be reduced by two-thirds when low-salinity brine was used as the solvent instead of high-salinity brine. The enhanced viscosity of polymers in low-salinity environments also reduces the risk of polymer precipitation and degradation, particularly in high-temperature reservoirs, extending the applicability of LSPF in challenging conditions.

The timing of polymer and low-salinity water injection is critical to maximizing the synergy between these two processes. Injecting polymers too early may result in inefficient oil mobilization due to insufficient wettability alteration, while injecting polymers too late may limit their effectiveness in stabilizing the displacement front. Akai et al. (2020)^37^ emphasized the importance of timing, noting that secondary low-salinity injection achieved better recovery due to more stable displacement fronts, whereas tertiary injections did not significantly impact oil recovery in stagnant regions of the reservoir.

This study aims to investigate the relative timing of polymer and low-salinity water synergies in enhancing oil recovery. By combining experimental data, pore-scale modeling, and digital rock analysis, this paper seeks to provide a comprehensive understanding of how the sequence and timing of these floods affect oil mobilization, sweep efficiency, and overall recovery. Understanding the interplay between polymer and low-salinity flooding at both the microscopic and macroscopic levels will help to optimize EOR strategies for a wide range of reservoir conditions.

### Problem statement

The relative timing of low salinity water injection and polymer flooding presents a complex and understudied challenge in enhanced oil recovery (EOR). The synergistic effects of these two techniques have demonstrated promising results individually, but the optimal sequencing and coordination of their application remain unclear. Experimental results suggest that Low salinity effect is mostly observed when the residual oil saturation, S_or_ is approached. Also, this is when water mobility is highest relative to oil mobility, so this might present the greatest advantage of polymer as a water mobility control agent. The lack of a comprehensive understanding of how the timing of low salinity and polymer injection influences oil recovery limits the efficiency and effectiveness of these EOR methods. Addressing this knowledge gap is crucial for developing robust strategies that maximize the synergies between low salinity and polymer flooding, ultimately enhancing oil recovery rates in diverse reservoir conditions.

## Methodology

### Pore network construction

This study utilizes a numerical Specialised Core Analysis Laboratory (*num*SCAL) to investigate the unsteady-state displacement of oil by low salinity (LS) brine and polymer solutions under varying injection sequences. The pore space is modelled as a network of capillary elements—pore bodies (nodes) connected by narrower pore throats (bonds). Each element is characterized by geometric parameters such as inscribed radius, length, volume, and shape factor. This idealized approach allows for both qualitative and semi-quantitative analysis of flow behavior at the pore scale.

#### Network types and topologies

Three network types are supported within the *num*SCAL simulator:

- **Bonds-Only Networks**: Simple cylindrical elements that transmit flow without representing pore body volume.
- **Pores-and-Throats Networks**: More realistic systems where pore bodies (nodes) are connected via narrower throats (bonds).
- **Extracted Micro-CT Networks**: Networks derived from high-resolution 3D micro-CT images of real rock samples, retaining true geometrical and topological features (e.g., Berea sandstone).

**Fig. 1:**
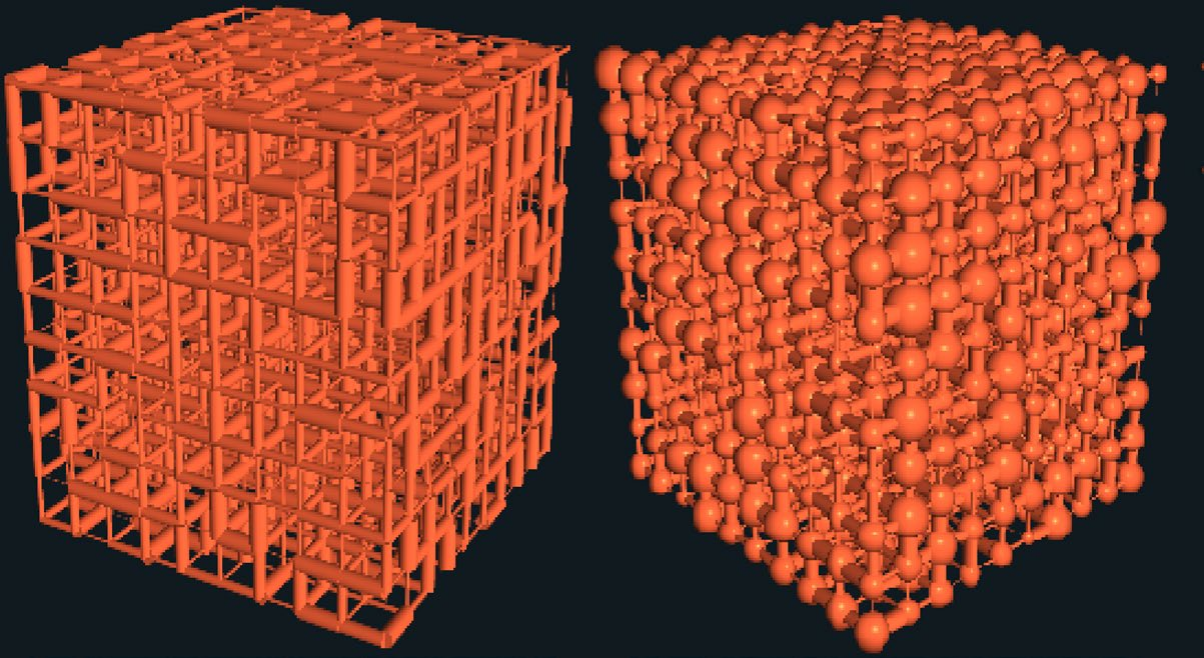
10 × 10 × 10 3D Bonds only model (left) and Pores and Throats model (right)) in numSCAL.

While our work is based primarily on 2D pore-and-throat networks for clarity and computational efficiency, the framework readily supports 3D modeling with minimal adjustments (Fig. 1).

Key parameters used to define the network include:

- **Network Dimensions (Nx × Ny × Nz)**: Defines the number of nodes along each axis. For 2D cases, Nz = 1.
- **Pore Size Distribution (PSD)**: Radii assigned randomly based on a statistically defined PSD to represent real reservoir heterogeneity.
- **Coordination Number (Z)**: Determines average connectivity per node; reduced artificially to simulate realistic heterogeneity.
- **Distortion Factor**: Adds positional noise to nodes to break geometric symmetry and mimic real rock variability.
- **Cross-Sectional Geometry**: Elements may have circular, triangular, or square sections, parameterized via the shape factor (G), which influences capillary entry pressure.

All results in this study are based on 2D pore network model simulations, chosen for their lower computational cost, quicker run times, and clearer visual interpretation compared to 3D models.

### Fluid flow Modeling – Unsteady-State drainage

We implement a dynamic, unsteady-state flow model that accounts for both capillary and viscous forces to model low salinity waterflooding and polymer flooding at the pore-scale. The governing assumptions in implementing the unsteady-state model are;

- The flow inside the capillary elements is considered to be laminar – the meniscus between two fluids is assumed to be always perpendicular to the axis of the bond.
- The fluids inside the capillaries are incompressible and immiscible.
- Film flow is modelled explicitly in the pores and throats model (oil layers and water films in the corners of angular elements).
- Counter-current imbibition against the main pressure gradient is not considered (elements exhibiting this are temporarily isolated).
- Ganglia mobilisation is not included in this model.
- Gravity forces are neglected.

Considering a scenario where water displaces oil in an oil-wet network, the simulation procedure is described thus;

1. **Initialization**: The network is populated with oil; initial water saturation (S_wi_) may be imposed.
2. **Capillary Pressure Evaluation**: At each element containing an oil/water interface, entry pressures are computed.
3. **Flow Field Solution**: Using mass conservation at nodes and incorporating capillary pressures, a global pressure field is determined.
4. **Counter-Current Throats Exclusion**: Elements exhibiting counter-current flow are removed iteratively to stabilize the pressure field.
5. **Flow Update**: Elemental flow rates are updated; volumes, phase positions, and saturations are recalculated.
6. **Iteration**: Steps 2–5 are repeated until the desired number of pore volumes is injected.

#### LS Brine and polymer effect modelling

Low salinity brine and polymer are modelled as miscible aqueous-phase with concentrations normalized between 0 and 1.

Correctly modelling the flow of LS water or polymer in the network takes the following conditions into consideration:

- LS water/polymer are injected into oil-wet systems (i.e., the waterfloods are drainage processes).
- LS water/polymer and HS water are miscible and mix instantly in a capillary element.
- No meniscus is considered between LS water/polymer and HS water.
- LS water and polymer cannot mix with oil or flow through the oleic phase.
- Fines migration during LS or polymer injection is not considered.
- Polymer adsorption, polymer rheology (shear thinning or thickening), and time-dependent LS wettability modification have been de-activated to simplify the interpretation of the polymer-LS synergy simulations (although these could be re-activated in future work).
- When LS water and polymer are flowing together, they are miscible, and no chemical interaction is modelled between them.

LS brine and polymer can flow from the inlet pores either immediately the simulation starts (secondary mode) or after some period of HS water injection (tertiary mode). We use a mass conservation law to update their corresponding concentrations according to the pressure field in the network. A similar approach was used in the work of Watson et al.. (2017)^18^ where the tracer evolution (an unsteady-state process) was coupled to a steady-state water injection model. In their simulations, the time required to achieve any steady-state volumetric change was estimated in order to update tracer concentrations in the network. Here, however, the aqueous phase dynamics are consistently tied to the unsteady-state drainage model and both the phase concentrations and phase saturations are updated dynamically at each iteration using the same time step.

**Fig. 2:**
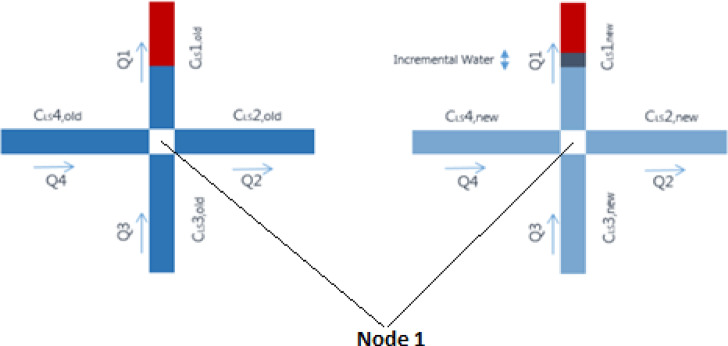
Phase configuration for four connected pores in a 2D network (left) at the start of a timestep (right) at the end of the timestep.

Consider the configuration shown by Fig. 2 and denote the LS brine concentration in each pore $\:i$ by $\:{C}_{i}$. The flow $\:{Q}_{i}$ in each pore is determined after solving the flow equations in the network, and the new volumetric water fractions $\:{F}_{w,new}$ are updated accordingly in all pores. Next, a new LS concentration $\:{C}_{LS,new}$ in pore 1, after a timestep Δt is computed after updating elementary water fractions in the network, and is calculated as follows:

1 $$\:{C}_{LS,new}=\:\frac{{F}_{w}{1}_{old}}{{F}_{w}{1}_{new}}\:{C}_{LS,1,old}+(\frac{{Q}_{1}}{{Q}_{into,1}}\:{\varPhi\:}_{LS,into,node}-{\varPhi\:}_{LS,outFrom1})\frac{\varDelta\:t}{{F}_{w}{1}_{new}{V}_{1}}$$

where V_1_ is the volume of pore 1 and $\:{Q}_{into,1}$ corresponds to the total volumetric flow into node 1. $\:{\varPhi\:}_{LS,into,node}$ refers to the mass flux of LS brine entering the node and is calculated as:

2 $$\:{\varPhi\:}_{LS,into,node}=\:{{Q}_{3}C}_{LS,3old}+\:{{Q}_{4}C}_{LS,4old}$$

We assume that the LS brine is partitioned among the downstream pores in proportion to their individual flows. Thus, the mass flux of LS entering pore 1 is given by $\:\frac{{Q}_{1}}{{Q}_{into,1}}$ $\:{\varPhi\:}_{LS,into,node}$. The mass flux of LS leaving pore 1 is calculated as:

3 $$\:{\varPhi\:}_{LS,outFrom1}=\:{{Q}_{1}C}_{LS,1old}$$

When polymer is flowing in the network, the concentration of polymer is computed in a similar fashion. Equation 1 remains valid when both LS brine and polymer are flowing, as no interaction between LS and polymer is considered.

The unsteady-state drainage model already described solves the pressure field in the network and updates the fluid fractions and phase saturations in the capillary elements. Then, the LS and polymer concentrations are updated based on mass conservation.

We assume that the main manifestation of the LS effect is wettability alteration – the contact angle associated with an element in the pore-scale network is modified when it comes into contact with LS brine. When an oil-filled capillary element comes into contact with low salinity water, we assume the contact angle changes towards a less oil-wet configuration, thus reducing the capillary resistance. We model this effect as a change of contact angle depending on salinity. The new contact angle is calculated as;

4 $$\:{\theta}_{new,i}=\:{\theta}_{initial,i}-\:\varDelta\:\theta\:x\:{{\rm\:H}}_{\theta\:}({C}_{LS},\:i)$$

where $\:\varDelta\:\theta$ is the maximum permitted contact angle change when LS concentration is equal to one and $\:{\rm\:H}$ refers to a Hill Function used to relate LS concentration to the magnitude of wettability change.

Polymer effect is modelled as a change in water viscosity. Depending on the concentration of polymer, the water viscosity is modified according to;

5 $$\:{\mu\:}_{new,i}=\:{\mu\:}_{HS}+\:\mu\:\:x\:{{\rm\:H}}_{\mu\:}\left({C}_{polymer,i}\right)$$

where $\:\varDelta\:\mu\:$ is the maximum change in viscosity when the concentration of polymer is 1, $\:{\mu\:}_{HS}$ is the high-salinity water viscosity while $\:{\rm\:H}$ refers to the Hill Function used to relate phase concentration to its corresponding effect.

A generalised Hill function is defined by;

6 $$\:{\rm\:H}\left({C}_{phase}\right)=\frac{{C}_{phase}^{n}(1-{\varGamma\:}^{n})}{{C}_{phase}^{n}\left(1-2{\varGamma\:}^{n}\right)+{\varGamma\:}^{n}}$$

where $\:\varGamma\:$ is the concentration at which half of ∆ϴ or $\:\varDelta\:\mu\:$ is reached, whilst n is the Hill function exponent that determines the strength of the phase response and $\:{C}_{phase}$ is the local concentration of each aqueous phase in each capillary element. In this woek we have set *n* = 30 and $\:\varGamma\:$ = 0.88 for both LS and polymer, meaning that the local salinity must be relatively low and the viscosity of the polymer solution must be high for a significant change to occur – of course these parameters can be modified in line with experimental data as and when such data becomes available.

### Frontal advance velocity

To characterize and compare flow behavior across different unsteady-state simulations, we define a *frontal advance velocity* as a reference metric. This parameter, which represents a form of Darcy velocity, is calculated using the following expression:.

7 $$\:\stackrel{-}{v}=\frac{{N}_{x}\:L\:Q}{V}$$

Where $\:\stackrel{-}{v}$ is the frontal advance velocity, $\:{N}_{x}$ is the number of nodes in the x-direction, $\:L$ is the length of each node, *Q* is the total flow rate at the inlet of the network and $\:V$ is the volume of all capillary elements, i.e., $\:V={\sum\:}_{i=0}^{n}{V}_{i}$ where n is the number of capillary elements.

The model considers two flooding modes: **secondary injection**, where the tracer (LS brine or polymer) is introduced at the start of the waterflood, and **tertiary injection**, where initial flooding is performed using high salinity (HS) water, and the tracer is introduced only after water breakthrough at the outlet.

We systematically explore various combinations of LS brine and polymer injection under both secondary and tertiary modes, with particular focus on the influence of injection sequence and timing on recovery performance and flow regime transitions.

### Simulation sequences and low Salinity – Polymer synergy

We will be investigating the impact of the timing and injection sequence of low salinity brine and polymer, whether they are injected simultaneously or phased in sequences to attempt to optimize oil recovery through the understanding of the driving mechanisms.

To study this, we have run a vast range of simulations covering a wide variation of injection protocols. We start by investigating the effect of LS brine and polymer separately and then go on to investigate their combined effect.

When simulating the injection of LS brine or polymer, two possible scenarios are considered:

- LS-sec, POL-sec: This refers to the injection of LS brine or polymer in secondary mode – injection of both occurs at the very beginning of the waterflood.
- LS-ter, POL-ter: This refers to the injection of LS brine or polymer in tertiary mode. Once a spanning HS water cluster forms following a period of HS water injection, a drop in pressure is observed at the system outlet, and no further significant recovery is usually observed. It is at this point that we start the injection of LS and/or polymer.

To simulate the LS effect on contact angle, we correlate the LS concentration to the contact angle change assigned to capillaries in direct contact with the LS brine. Initially, all the capillary elements are considered oil-wet and assigned a contact angle equal to 140^o^. As the concentration of LS brine starts to build up in a capillary element, the associated contact angle starts to decrease until it reaches a minimum value corresponding to a LS brine concentration equal to one (note that a high concentration of LS brine corresponds to a *low* value of salinity). We consider a scenario that corresponds to a change from a strongly oil-wet configuration to a neutral-wet configuration. The contact angle change is Δθ = 45^o^ and a shift from 140^o^ to 95^o^ is expected in the contact angles of the capillaries in direct contact with high concentrations of low salinity brine.

The effect of polymer on water viscosity is simulated in a similar fashion. We correlate the polymer concentration to the water viscosity in the corresponding capillary element. Initially, the HS water viscosity is set to 1cP and polymer enters each element at the maximum value of viscosity (as the polymer concentration is equal to one). In this study, water viscosity increases from 1cP to 10cP as polymer concentration approaches one: this effectively shifts the viscous ratio M (=µ_oil_/µ_polymer_) from 10 to 1 if we are injecting water into oil with viscosity of 10cP.

The various injection sequences are considered in this work are enumerated below:

**LS brine injection**:

1. Inject high salinity (HS) water to S_or_ then replace with LS brine (tertiary injection).
2. Inject HS water to an intermediate saturation, then replace with LS brine (still secondary injection, but preceded by high salinity formation water).
3. Inject LS brine in secondary injection mode.

**LS brine and Polymer Simultaneously**.

4. Inject HS water to S_or_ then replace with LS brine and polymer *simultaneously* (tertiary injection).
5. Inject HS water to an intermediate saturation, then replace with LS brine and polymer injected *simultaneously* (still secondary injection, but preceded by high salinity formation water).
6. Inject both LS brine and polymer simultaneously in secondary injection mode.

**Polymer injection AFTER LS brine**.

7. Inject HS water to S_or_ and then flood with several PVs of LS brine. Then inject polymer *simultaneously* with LS brine.
8. Inject HS water to an *intermediate* saturation and then flood with several PVs of LS brine. Then inject polymer *simultaneously* with LS brine.
9. Inject LS brine in secondary injection mode followed by the injection of polymer *simultaneously* with LS brine.

**Polymer injection BEFORE LS brine**.

10. Inject HS water to S_or_ and then flood with polymer for several PVs. Then inject LS brine *simultaneously* with polymer.
11. Inject HS water to an *intermediate* saturation and then flood with polymer for several PVs. Then inject LS brine *simultaneously* with polymer.
12. Inject polymer in secondary injection mode followed by the injection of LS brine *simultaneously* with polymer.

*S*_*or*_ *defined here is* $\:{S}_{or}^{HS}$, *which is the residual oil saturation after high salinity (HS) water injection and is the point where any additional water injected will not result in further recovery of oil.*

$\:{S}_{intermediate}^{HS}$ *is a saturation before breakthrough of HS water.*

### Model limitation

While pore network modeling offers valuable insight into pore-scale displacement processes, several limitations must be acknowledged. The simplified geometry and reduced dimensionality (2D in this study) may not fully capture the complexity of real reservoir rock structures, including heterogeneities in pore connectivity and three-dimensional flow pathways. Assumptions such as incompressible flow, absence of ganglia mobilization, and neglect of gravity or fines migration can lead to deviations from actual reservoir behavior.

We acknowledge that absolute recovery values predicted by our model may exceed those commonly reported in coreflood experiments or field trials. However, the goal is not to replicate absolute experimental recovery, but rather to isolate and highlight relative improvements due to the timing of LS and polymer injection. These improvements are expressed relative to a high-salinity (HS) waterflood base case under idealized, controlled conditions—assuming no crossflow, no fracture-induced bypassing, and uniform injection.

Although ganglia mobilization is not explicitly modeled, the framework captures changes in phase connectivity and occupancy through capillary pressure thresholds and viscosity contrasts. The observed recovery gains are attributed to:

- Improved pore accessibility from LS-induced wettability alteration.
- Suppression of viscous fingering by early polymer injection.
- More stable displacement fronts, as indicated by Regime Index trends.

These mechanisms have been validated in micromodel studies and CT imaging (e.g., Zhang et al., 2024)^21^, supporting the relevance of modeled trends despite differences in absolute values.

Additionally, the model treats polymer and LS brine as idealized tracers, neglecting chemical interactions, adsorption, or non-Newtonian rheology—factors that introduce uncertainty in field-scale extrapolation. Numerical artifacts, discretization choices, and simplified wettability alteration models may further contribute to discrepancies. Despite these constraints, the model remains a robust tool for exploring mechanistic behavior and evaluating EOR strategies in a reproducible digital environment.

## Results and discussion

### Base case data

In this study, we use a 2D distorted pores and throats network. As a base case, we consider the networks to be initially oil-wet with contact angles equal to 140^o^. The initial viscous ratio is equal to 10 ($\:{\mu\:}_{water}$= 1cP, $\:{\mu\:}_{oil}$ = 10cP), and water is injected with an average frontal advance velocity V equal to 1 m/day. LS brine gradually shifts the contact angles from 140^o^ to 95^o^, whilst polymer modifies water viscosity from 1cP to 10cP. Table 1 summarises the network properties and flow parameters used in the base case study.

### Seed number

The 2D statistically generated networks used in this study sample pore radii from a predefined pore size distribution. A random seed is a parameter used by random number generators to ensure that whenever we sample a sequence of numbers from a random distribution, we always end up with the same sequence – this can be useful when wanting to compare different flooding sequences on an identical network. However, running a single simulation using a single seed cannot be considered a very reliable approach to quantify the effect of a particular flooding protocol and so we run multiple simulations for each scenario by using multiple seeds. In this study, every scenario has been carried out using 5 different networks that share the same average properties, but which differ on a local scale. The results are consequently presented as statistical distributions rather than single data points.

**Table 1:** Base case data for the LS Brine and polymer synergy.

Parameter	Value
Network size	200 × 100
Coordination number, Z	4
Degree of Distortion	0.1
Average Pore Length	100 μm
Pore Size Distribution	Uniform
Aspect Ratio	1.5
Inscribed Radius (Min, Max)	(1,30) µm
Initial wettability	Oil-Wet (ϴ = 140^o^)
Initial water saturation	0.0
Interfacial Tension	20 N/m (dynes/cm)
Oil viscosity	10cp
Water viscosity	1cp
Polymer solution viscosity	10cp
Frontal advance velocity	1 m/day, 10 m/day

### Model results

To explain some of the observations, we will make use of the **Regime index**, $\:{R}^{index}$ which measures the dominant force during a flood: $\:{R}^{index}>0$ implies viscous perturbation and $\:{R}^{index}<0$ implies capillary dominance. Viscous dominance usually begins somewhere around $\:{R}^{index}>1$ meaning that the viscous force is at least twice the capillary resistance. For $\:0<{R}^{index}<1$, there is a transition between capillary and viscous regimes. $\:{R}^{index}$ is a useful indicator and is calculated at each timestep by the equations:

8 $$\:\stackrel{-}{{P}_{c}}=\frac{1}{n}\sum_{i=1}^{n}2\sigma\:\text{cos}\theta\:/{R}_{i}$$

9 $$\:{R}^{index}=(\varDelta\:P-{\stackrel{-}{P}}_{c})/{\stackrel{-}{P}}_{c}$$

where n is the current number of water filled elements, R_i_ is the radius of water filled element *i*, $\:\sigma\:$ is the oil-water interfacial tension, $\:\theta\:$ is the contact angle, $\:\stackrel{-}{{P}_{c}}$ is the current mean capillary pressure in the network and $\:\varDelta\:P$ is the current pressure gradient across the network.

#### LS Brine injection

We begin by comparing the first injection sequence where the following cases are considered (i) High salinity water is injected for 3PVs (ii) high salinity (HS) water is first injected to S_or_ and then replaced by LS brine till a total of 3PVs injected (iii) HS water is injected to an intermediate saturation and then replaced with LS brine for a total of 3PVs (iv) LS brine is injected in secondary mode for 3PVs.

For a **mobility ratio** of 10 and **frontal advance velocity** of 1 m/day and the base network data presented in Table 1, $\:{\varvec{S}}_{\varvec{o}\varvec{r}}^{\varvec{H}\varvec{S}}$ is reached at approximately 0.65PVs injected and breakthrough of HS water occurs after 0.35PV.

**Fig. 3:**
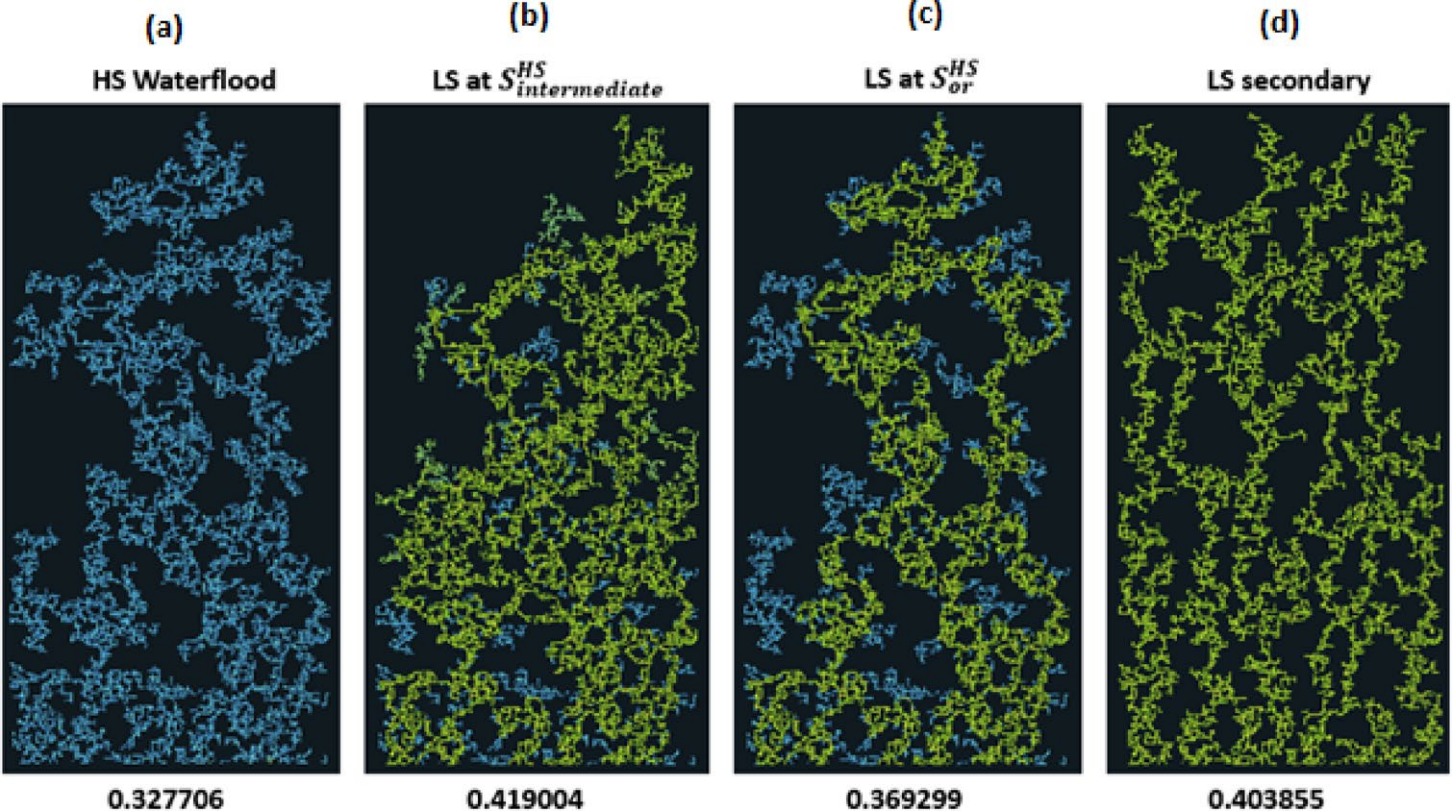
Water distribution after 3PVs injected, M = 1, v = 1 m/day. From Left to right: **(a)** HS water flood **(b)** HS water replaced with LS brine after several PVs before breakthrough of HS water **(c)** HS water replaced with LS brine after residual saturation of oil is reached **(d)** Secondary injection of LS brine.

**Fig. 4:**
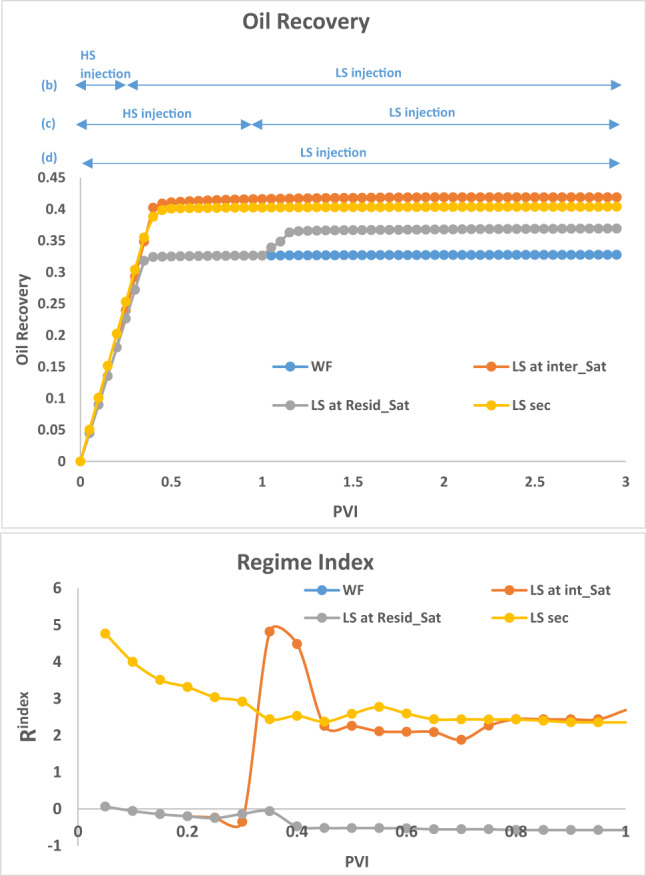
Oil recovery and Regime index plots after 3PVs injected for HS waterflood and LS brine injection for Sequence 1–3.

The results in Fig. 3, can be interpreted by examining the **R**^**index**^ in Fig. 4. For the HS water waterflood (Fig. 3(a)), the displacement process is capillary dominated, giving rise to large clusters of bypassed oil due to capillary resistance and the pressure gradient being unable to displace extra oil. When LS brine is injected after 1PV of HS brine (Fig. 3(c)) (which is after residual oil saturation, $\:{\varvec{S}}_{\varvec{o}\varvec{r}}^{\varvec{H}\varvec{S}}$, has been reached), the injected LS brine travels along existing HS pathways and is only able to displace some additional oil from its direct neighbours, with most of the flow being through established pathways.

The LS brine is still able to affect the capillary entry pressure of its directly connected oil-filled neighbours but is unable to greatly influence the pressure distribution, which is why the regime index is the same as that seen in the HS waterflood. This flood yields an additional 4% oil recovery. When LS brine is injected at an intermediate saturation (0.25PVs, Fig. 3(b)) we see a change in regime index starting from around 0.3PV and the subsequent shift to a viscous dominated displacement. Also, the displacement is more stable when LS brine is injected at an intermediate saturation before breakthrough. This limits the capillary fingering that would have developed without LS brine injection. The oil recovery plot shows that while the recovery for HS water plateaued at approximately 0.3PVI, the recovery from LS brine injection initiated at an intermediate HS saturation continued to rise with ~ 10% more oil being recovered until it plateaued at 0.45PVI. When LS brine is injected in secondary mode (Fig. 3(d)), the displacement is viscous dominated from the start of injection, and we observe a viscous fingering pattern in the displacement due to the change in contact angle (wettability). Although smaller pore sizes are now accessible to the displacing phase, this change in regime and the elongation of the water clusters leads to more bypassed oil and recovery is not optimised.

**Fig. 5:**
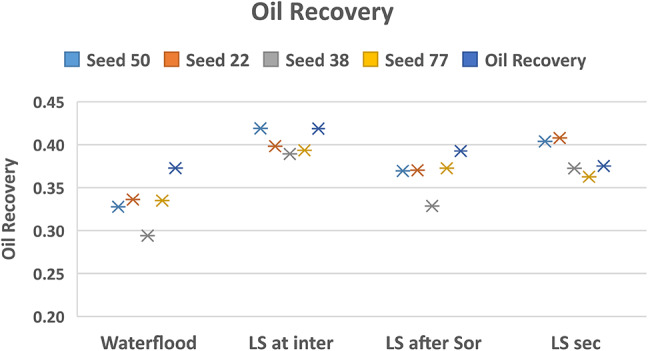
Oil recovery for injection sequences 1–3 and HS waterflood after 3PVs injected for M = 10 across 5 different capillary networks, v = 1 m/day.

To confirm the observation of a more stable displacement pattern when LS brine is injected following an intermediate HS saturation before breakthrough, we repeat the simulation for 5 different networks represented by different seed numbers. This is shown in Fig. 5.

The results for the different network structures confirm that the benefit of LS brine injection is impacted by the timing of the injection: we see better performance when LS brine is injected in general but maximising the recovery potential might require injecting LS brine before breakthrough of HS brine, rather than injecting LS brine from the start of the water flood.

This can be explained by the observation that, in secondary injection of LS, the capillary forces are altered at the start of the waterflood and the flow regime is unstable viscous fingering, oil is bypassed in the process and flow takes the shortest paths to the outlet. When LS brine is injected at an intermediate HS saturation, the flow is initially capillary dominated and the brine is better connected in the transverse direction: the subsequent introduction of LS brine to alter the wettability and reduce capillary resistance means the LS brine has access to more areas of the network and can advance in a more stable way, displacing more oil in the process.

#### LS Brine and polymer simultaneously

**Fig. 6:**
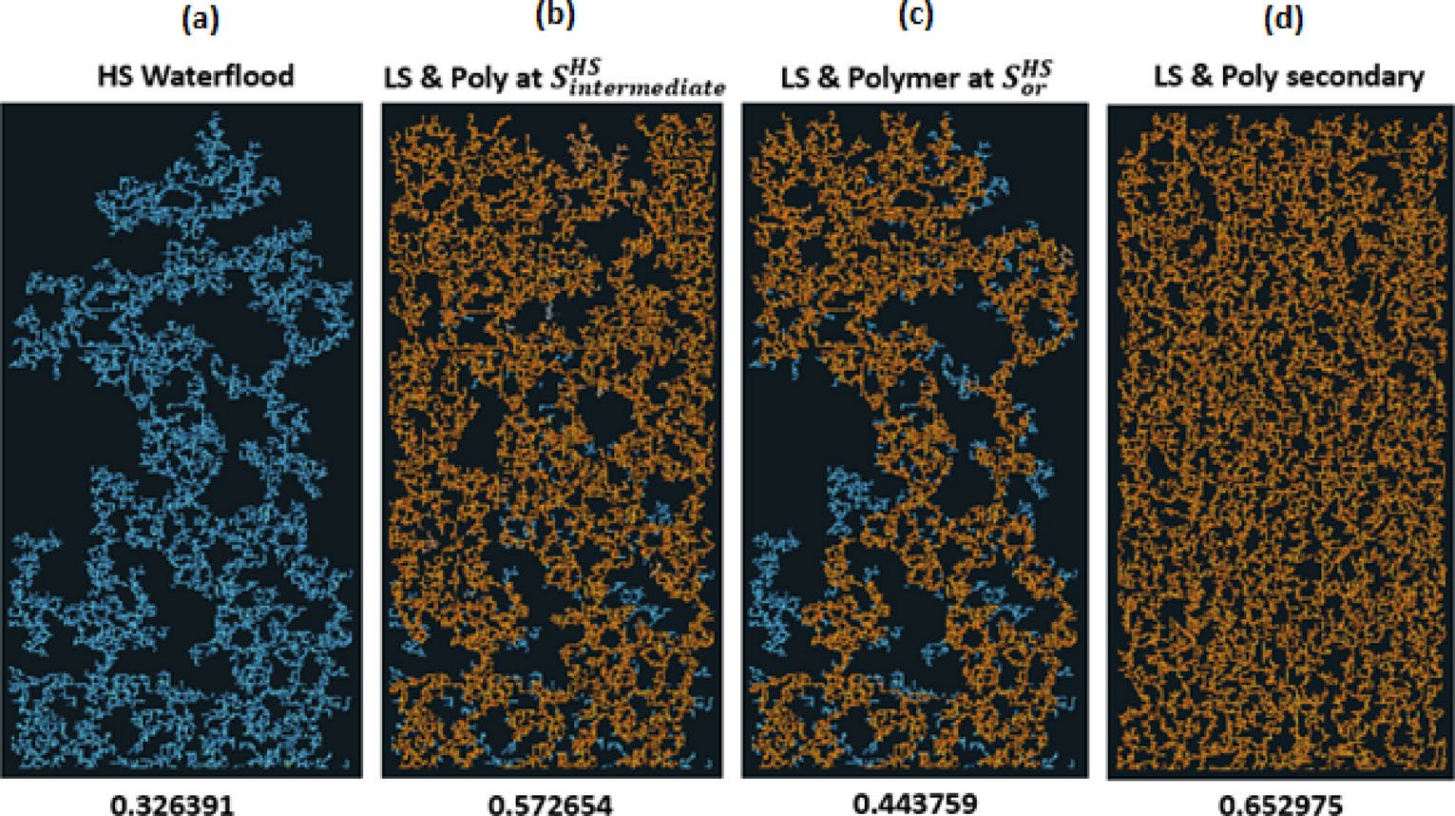
Water distribution after 3PVs injected for M = 10, v = 1 m/day. From Left to right: **(a)** HS water flood for 3PVs **(b)** HS water is injected to an intermediate saturation, then replaced with LS brine and polymer injected simultaneously **(c)** HS water is injected to Sor then replaced with LS brine and polymer simultaneously **(d)** LS brine and polymer simultaneously injected in secondary mode.

If we consider the previous sequences, but now with polymer injected simultaneously with LS brine after $\:{\varvec{S}}_{\varvec{o}\varvec{r}}^{\varvec{H}\varvec{S}}$, at $\:{\varvec{S}}_{\varvec{i}\varvec{n}\varvec{t}\varvec{e}\varvec{r}\varvec{m}\varvec{e}\varvec{d}\varvec{i}\varvec{a}\varvec{t}\varvec{e}}^{\varvec{H}\varvec{S}}$ and with LS brine in secondary mode, the final distribution is shown in Fig. 6.

The simultaneous injection of LS brine and polymer in general gives a better recovery than LS brine injection on its own. For the case of LS brine and polymer injection after $\:{\varvec{S}}_{\varvec{o}\varvec{r}}^{\varvec{H}\varvec{S}}$ the displacement follows the existing pathways established by the HS water flood for much of the injection but recovers some additional oil from the directly connected neighbours after the reduction in the capillary entry pressure (due to LS effect and the increased pressure gradient from the polymer injection). This yields an increase of 12% in comparison to the HS waterflood, and more than 7% in comparison with only LS brine injection at this point.

**Fig. 7:**
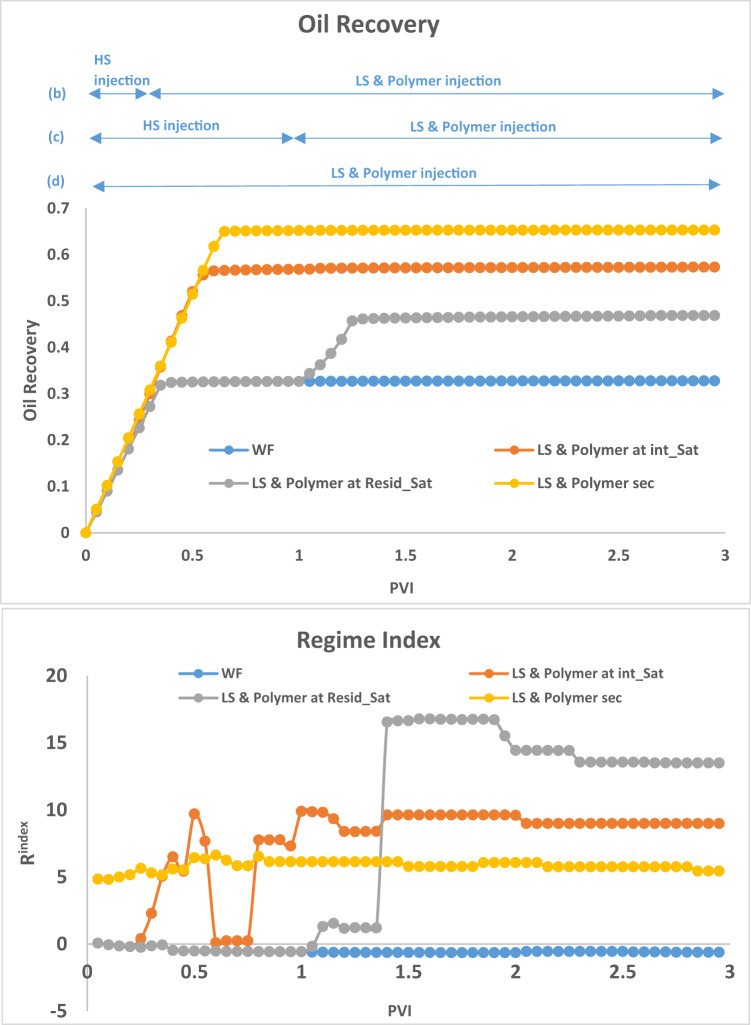
Oil recovery and Regime index plots for HS waterflood and injection sequences 4–6 after 3PVs injected M = 10, v = 1 m/day.

For LS brine and polymer injection at $\:{\varvec{S}}_{\varvec{i}\varvec{n}\varvec{t}\varvec{e}\varvec{r}\varvec{m}\varvec{e}\varvec{d}\varvec{i}\varvec{a}\varvec{t}\varvec{e}}^{\varvec{H}\varvec{S}}$ (Fig. 6(b)), although LS brine injection on its own was able to displace additional oil, with polymer, the injected fluid can access even more areas of the network, displacing more oil in the process. The regime index plot (Fig. 7) shows a switch to a viscous dominated regime after the introduction of both LS brine and polymer, although the regime index reduces before increasing again due to some fingers breaking through. This suggests that injecting polymer with LS brine *before* $\:{\varvec{S}}_{\varvec{o}\varvec{r}}^{\varvec{H}\varvec{S}}$ yields a much better performance because after $\:{\varvec{S}}_{\varvec{o}\varvec{r}}^{\varvec{H}\varvec{S}}$, water is most mobile and is the only fluid flowing in the network – the stabilizing effect of polymer on the water is therefore not maximised in relation to displacing oil because the oil is already immobile. However, injecting both polymer and LS in secondary mode yields the best recovery with 65% oil recovered. This can be explained by the water fingers being stabilized, reducing the effect of viscous fingering on bypassed oil. LS is also able to access more areas of the network due to the higher viscous forces induced by the polymer, with 10cp polymer solution replacing 10cp oil, rather than 1cp LS brine replacing 10cp oil.

**Fig. 8:**
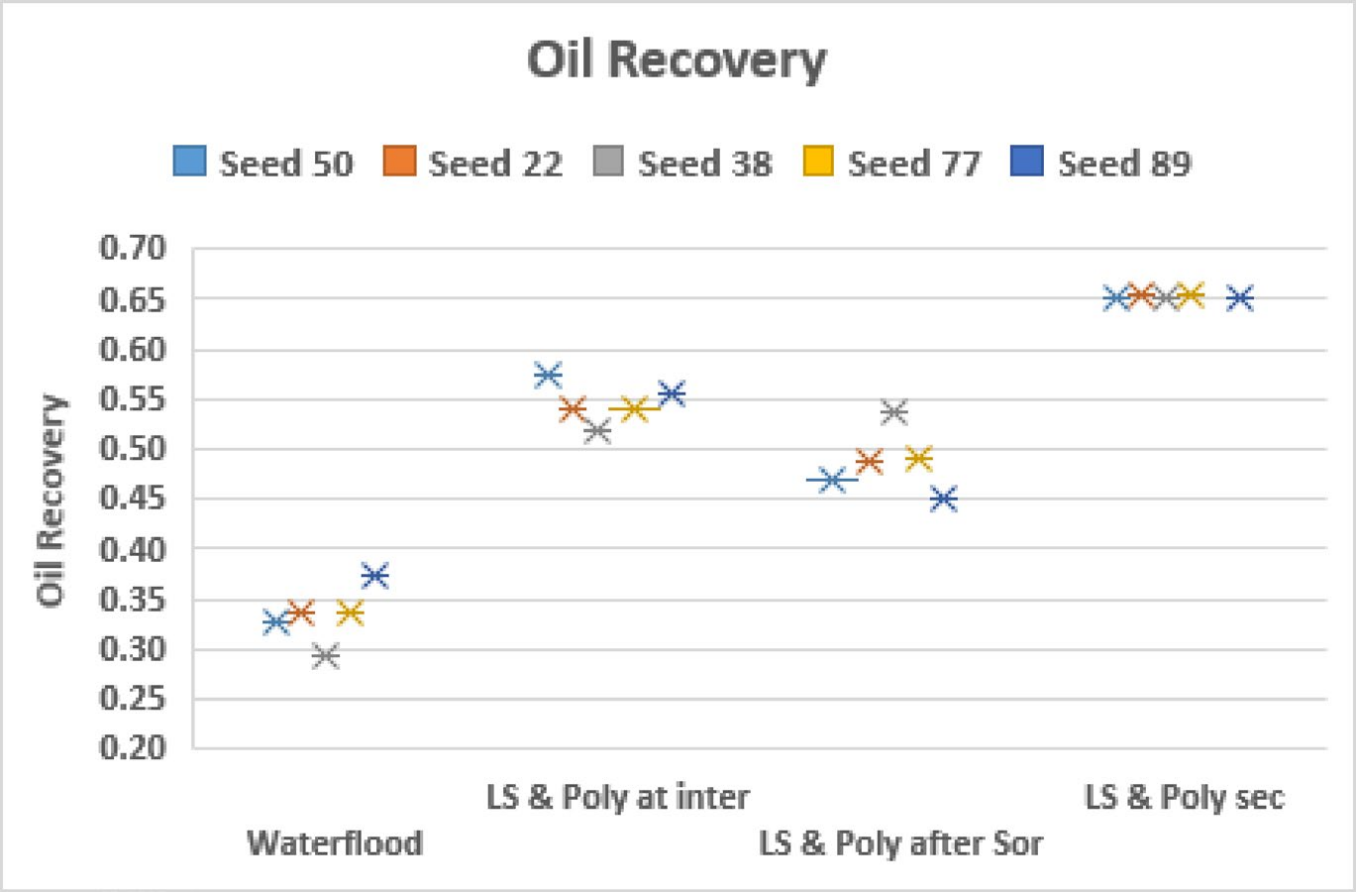
Oil recovery for injection sequences 4–6 and HS waterflood after 3PVs injected for M = 10 across 5 different capillary networks, v = 1 m/day.

Figure 8 confirms the observations from Fig. 6 with the simultaneous injection of LS brine and polymer recovering the most oil, irrespective of seed number. We also conclude that simultaneous LS brine and polymer injection at $\:{\varvec{S}}_{\varvec{i}\varvec{n}\varvec{t}\varvec{e}\varvec{r}\varvec{m}\varvec{e}\varvec{d}\varvec{i}\varvec{a}\varvec{t}\varvec{e}}^{\varvec{H}\varvec{S}}$ generally performs better than LS brine and polymer injection at $\:{\varvec{S}}_{\varvec{o}\varvec{r}}^{\varvec{H}\varvec{S}}$.

#### Polymer injection AFTER LS Brine

**Fig. 9:**
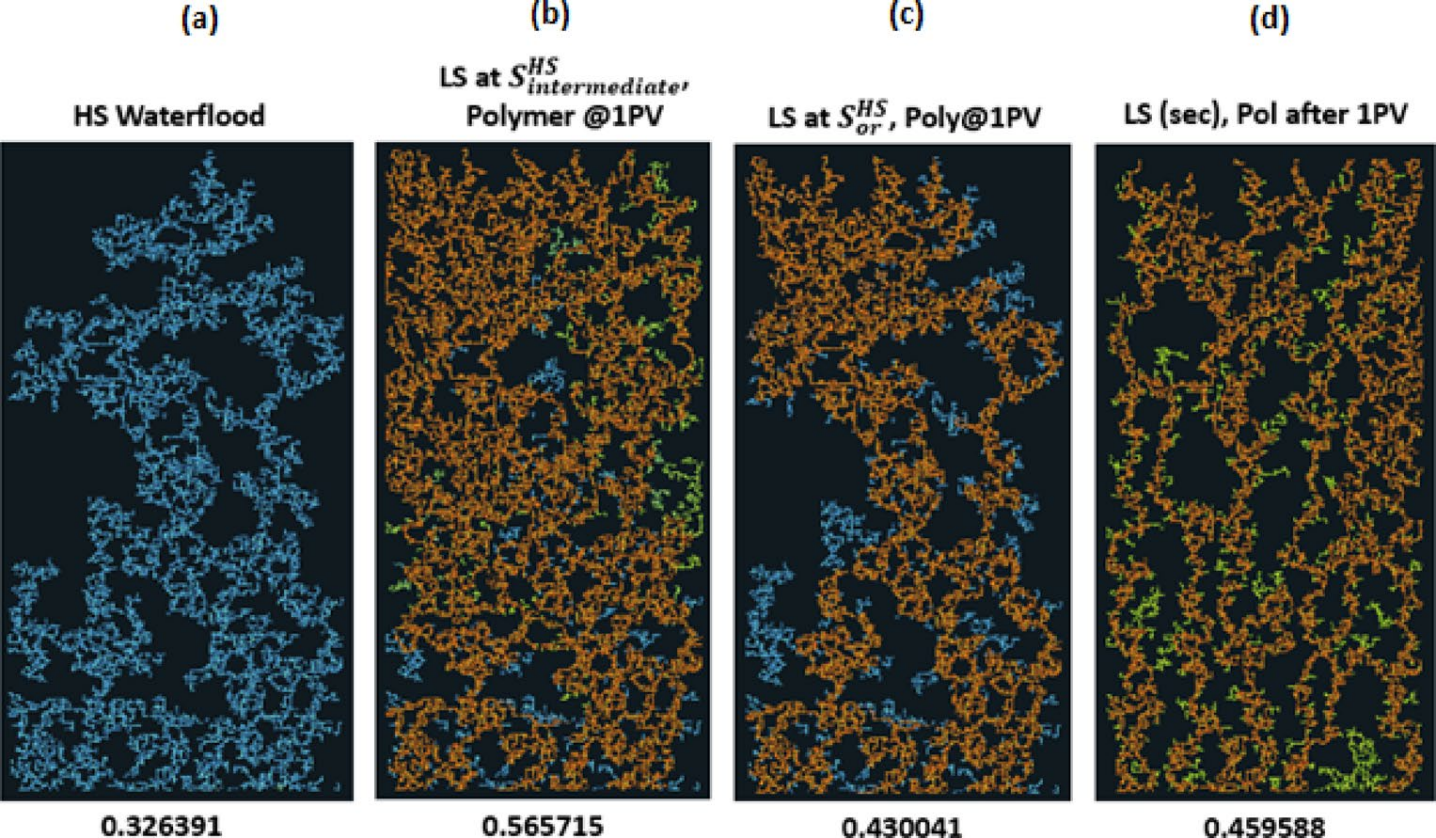
Final distribution after 3PVs injected for M = 10, v = 1 m/day with LS brine injection preceding polymer injection. From Left to right: **(a)** HS water flood for 3PVs **(b)** HS water is injected to an intermediate saturation and then flooded with LS brine for a total of 1PV. Then polymer is injected simultaneously with LS brine for a further 2PVs **(c)** HS water is injected to Sor and then flooded with LS brine for a total of 1PV. Polymer is then injected simultaneously with LS brine for a further 2PVs **(d)** LS brine is injected in secondary mode for 1PV followed by the simultaneous injection of polymer and LS brine for a further 2PVs.

The Fig. 9 shows the comparison where we inject LS brine *before* polymer. Polymer is injected after 1PV of HS water and/or LS brine injection.

**Fig. 10:**
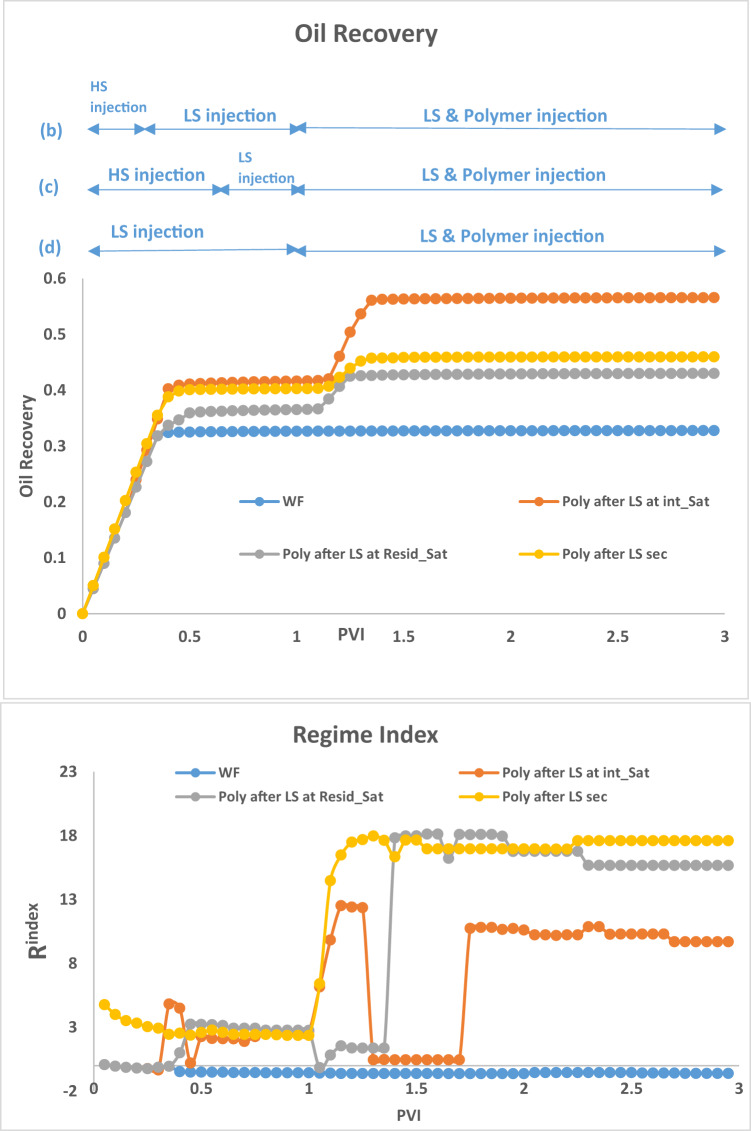
Oil recovery and Regime index plots for HS waterflood and injection sequences 7–9 after 3PVs injected. M = 10, v = 1 m/day.

In Fig. 9 polymer is injected at 1PV in all 3 cases, while the timing of LS brine injection is varied from the beginning of the water flooding to an intermediate saturation of HS water and finally to residual HS water saturation.

The observations in Fig. 9 are consistent with the observations in Fig. 3 regarding injecting LS brine at $\:{\varvec{S}}_{\varvec{i}\varvec{n}\varvec{t}\varvec{e}\varvec{r}\varvec{m}\varvec{e}\varvec{d}\varvec{i}\varvec{a}\varvec{t}\varvec{e}}^{\varvec{H}\varvec{S}}$. With LS brine injection at $\:{\varvec{S}}_{\varvec{i}\varvec{n}\varvec{t}\varvec{e}\varvec{r}\varvec{m}\varvec{e}\varvec{d}\varvec{i}\varvec{a}\varvec{t}\varvec{e}}^{\varvec{H}\varvec{S}}$ we observe from the regime index plot (Fig. 10) that the flow regime changes after LS brine is injected and from the water distribution in Fig. 9 we also observe the displacement is more stable, leading to additional recovery. This allows the polymer injected at 1PV to recover a significant amount of oil in the network (17%) because the polymer can now access more areas of the network where the LS brine effect has modified the capillary entry pressures favourably and polymer provides the pressure gradient required to drive recovery of oil from these areas of the network. The polymer also stabilises the frontal advance even further. This again confirms our observations that injecting LS brine at an intermediate saturation of HS water (i.e., before HS breakthrough) yields better recovery.

When LS brine is injected in secondary mode i.e., at the start of the waterflood, the capillary pressure field is altered as wettability is modified by the injected LS brine and this results in an unstable viscous fingering displacement. This is also evidenced by the Regime index in Fig. 10 where the flow regime is viscous dominated while the others are capillary dominated from the start of the waterflood. Because of the unstable displacement and fingers that have evolved, the impact of polymer after 1PV for a further 2PVs is only able to displace 5% additional oil in this case because the flow continues longitudinally towards the outlet with transverse flow across the network limited due to viscous fingering.

**Fig. 11:**
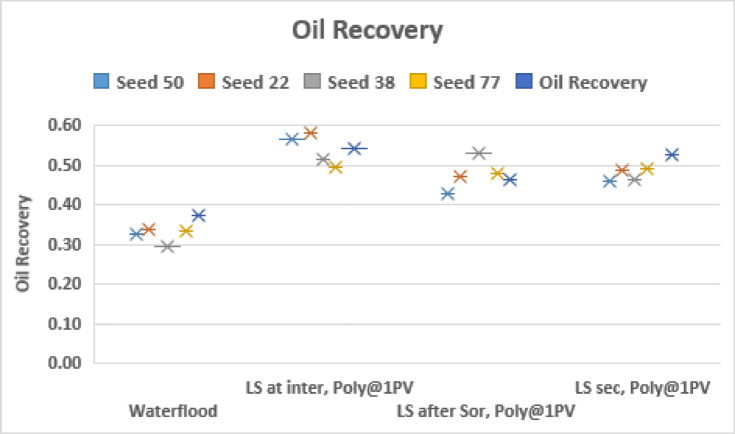
Oil recovery for injection sequences 7–9 and HS waterflood after 3PVs injected for M = 10 across 5 different capillary networks, v = 1 m/day.

The results of Fig. 11 show that when LS brine is injected before polymer solution, the recovery is optimized when the LS brine is injected at an intermediate saturation of HS water.

#### Polymer injection BEFORE LS Brine

**Fig. 12:**
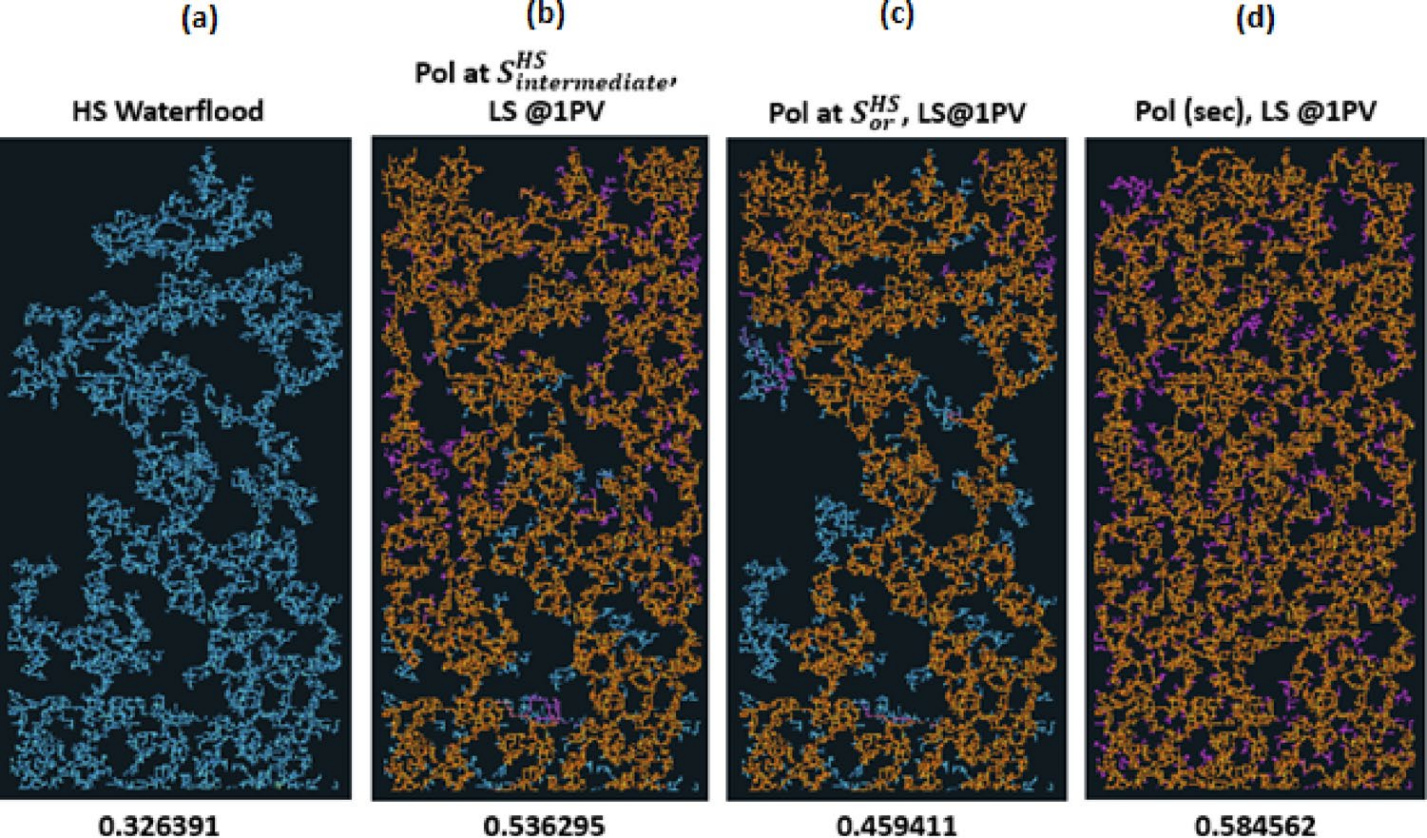
Final distribution after 3PVs injected for M = 10, v = 1 m/day with Polymer injected prior to LS brine. From Left to right: (a) HS water flood for 3PVs (b) HS water is injected to an intermediate saturation and then flooded with polymer for a total of 1PV. LS brine is then injected simultaneously with polymer for a further 2PVs. (c) HS water is injected to Sor and then flooded with polymer for a total of 1PV. Then LS brine is injected simultaneously with polymer for a further 2PVs. (d) polymer is injected in secondary mode for 1PV, followed by the injection of LS brine simultaneously with polymer for a further 2PVs.

If we change the order and inject polymer before LS, the distribution is shown in Fig. 12.

**Fig. 13:**
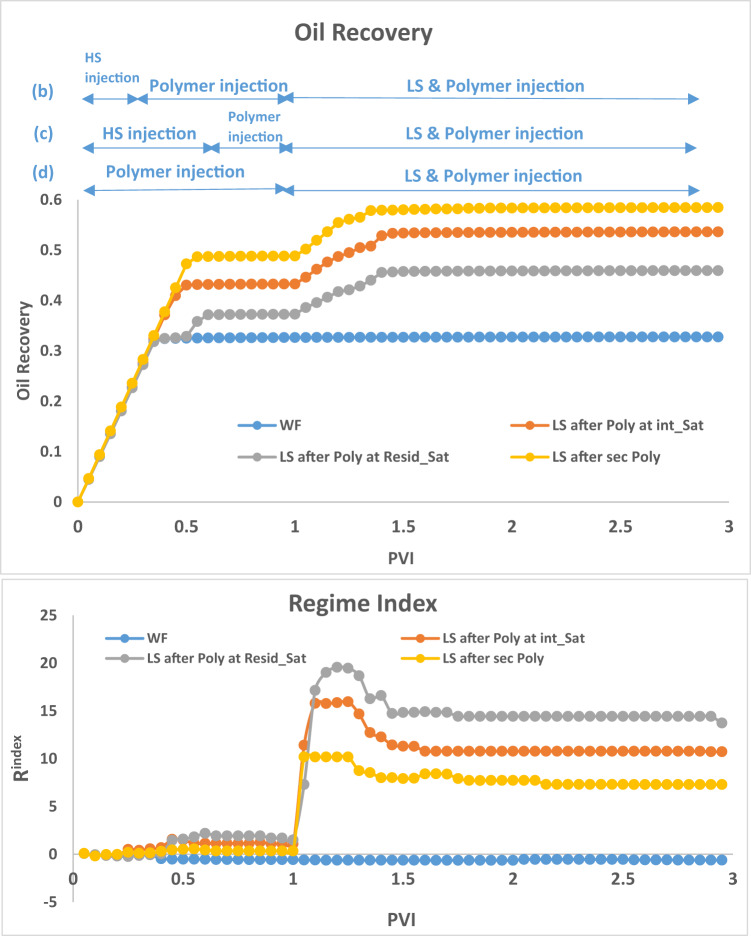
Oil recovery and Regime index plots for HS waterflood and injection sequences 10–12 after 3PVs injected. M = 10, v = 1 m/day.

The plots of oil recovery in Fig. 13 clear shows the impact of timing of polymer injection on the amount of oil recovered. The earlier the injection of polymer, the more the oil recovered. *Subsequent injection of LS brine yielded approximately 10% additional oil in all 3 cases*. This contrasts the observations when LS brine was injected before polymer.

When polymer is injected in secondary mode (Fig. 12(d)), the polymer travels as a stabilized front and has a better areal sweep efficiency and after the introduction of LS brine, capillary resistance is reduced in the higher capillary entry pressure pores that polymer on its own could not displace, causing the fingers to thicken and swell.

With the introduction of polymer at residual saturation of HS water, $\:{\varvec{S}}_{\varvec{o}\varvec{r}}^{\varvec{H}\varvec{S}}$, the polymer solution mostly travels through established pathways as has been the case with the previous sequences where LS injection was initiated at $\:{\varvec{S}}_{\varvec{o}\varvec{r}}^{\varvec{H}\varvec{S}}$ – only a few fingers become extended and some additional oil is displaced when LS brine is subsequently introduced, predominantly through the lateral expansion of polymer fronts and improved access to bypassed zones. When polymer is injected at $\:{\varvec{S}}_{\varvec{o}\varvec{r}}^{\varvec{H}\varvec{S}}$ and LS brine is injected afterwards at 1PV, the oil recovery is higher than when LS brine is injected at $\:{\varvec{S}}_{\varvec{o}\varvec{r}}^{\varvec{H}\varvec{S}}$ and polymer is injected afterwards at 1PV.

From the observations here, it can be concluded that the injection of LS brine after polymer injection causes the thickening and swelling of fingers leading to additional oil recovery.

The regime index plot in Fig. 13 shows the switch to a viscous dominated regime after LS brine is injected at 1PV due to the reduction in capillary entry pressures, confirming our earlier observations.

**Fig. 14:**
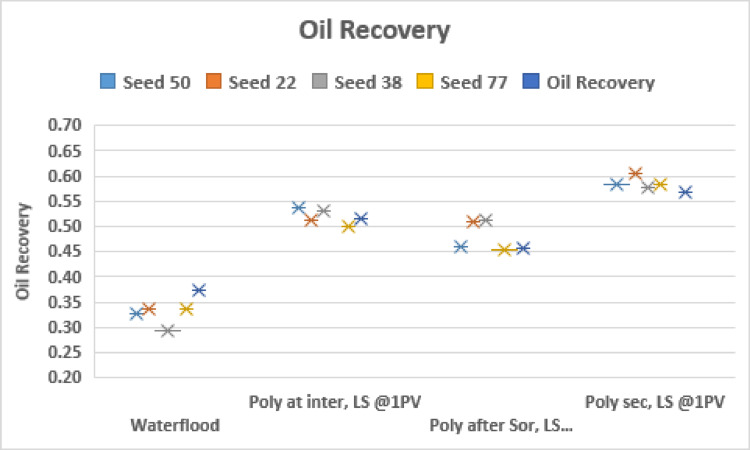
Oil recovery for injection sequences 10–12 and HS waterflood after 3PVs injected for M = 10 across 5 different capillary networks, v = 1 m/day.

From Fig. 14 we can see that the earlier the polymer injection, the more the oil recovered – this is independent of seed number.

**Fig. 15:**
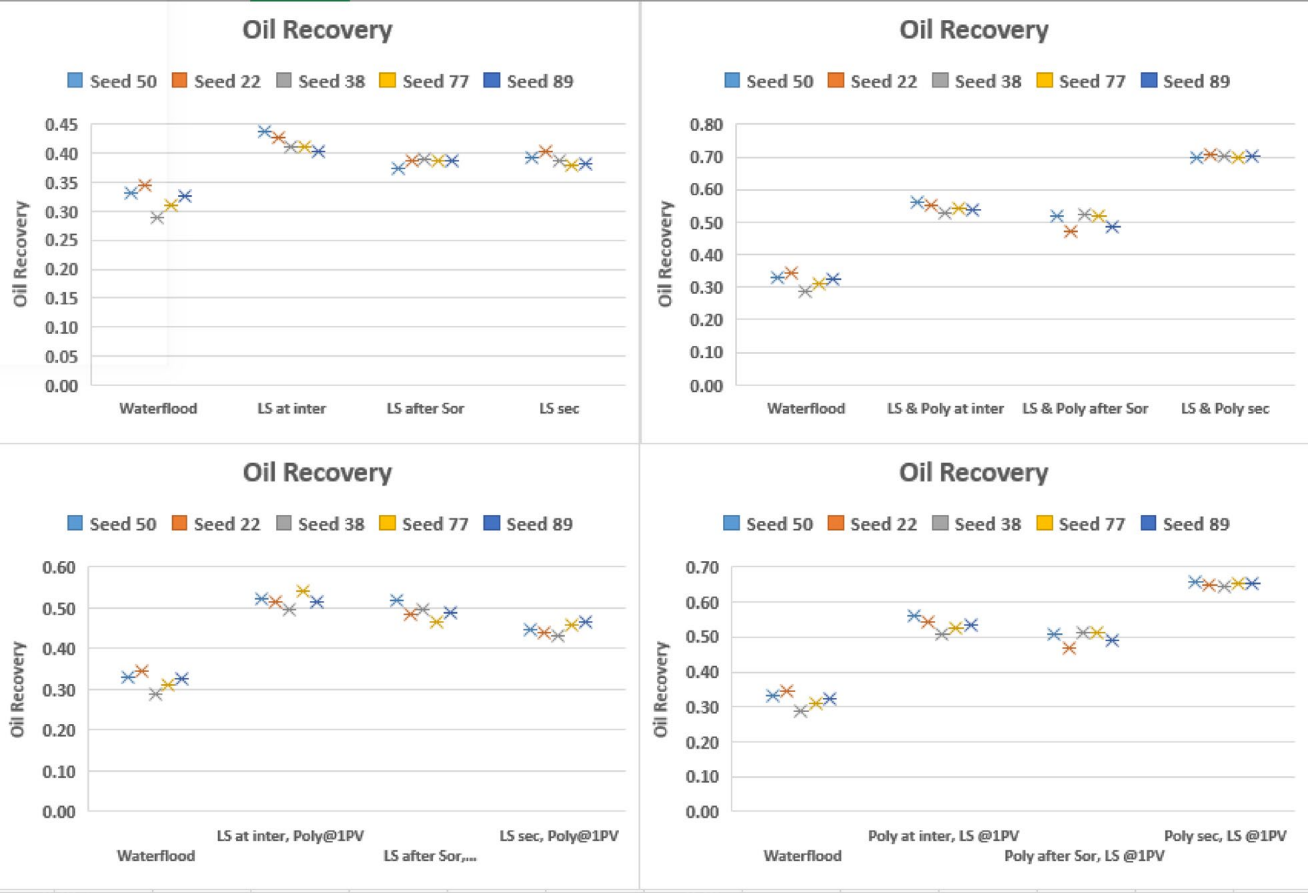
Oil recovery for all injection sequences 1–12 and different seed numbers at 10 m/day frontal advance velocity, M = 10.

Finally, we will examine the results of oil recovery for the injection sequences considered thus far at **10 m/day**. Results are shown in Fig. 15.

At 10 m/day, When LS brine is injected alone, the results are very similar for secondary LS brine injection and LS brine injection at $\:{\varvec{S}}_{\varvec{o}\varvec{r}}^{\varvec{H}\varvec{S}}$ and LS injection at $\:{\varvec{S}}_{\varvec{i}\varvec{n}\varvec{t}\varvec{e}\varvec{r}\varvec{m}\varvec{e}\varvec{d}\varvec{i}\varvec{a}\varvec{t}\varvec{e}}^{\varvec{H}\varvec{S}}$. This is because the high flow rate injection shifts the flow regime to viscous dominated and the impact of capillary pressure is minimized.

When LS brine is injected before polymer, secondary LS brine injection consistently produced the lowest oil recovery across all networks because the viscous fingering at 10 m/day is worse than at 1 m/day, leaving significant oil behind.

When polymer is injected in secondary mode, either together with LS brine or prior to LS brine, the recovery is significantly higher than at 1 m/day.

### Experimental validation

To complement and validate the findings on the impact of relative timing between low salinity (LS) brine and polymer injection on oil recovery, a range of core flooding and micromodel experiments from the literature provide strong empirical evidence. These experimental studies offer crucial insights into how wettability alteration, capillary entry pressure reduction, and mobility control mechanisms interact at both the pore scale and laboratory core scale. They also serve to bridge the gap between digital simulation tools and field-level design by physically replicating fluid dynamics in porous media under controlled conditions.

Several studies have demonstrated the critical influence of injection sequence on oil recovery. For instance, Al-Saedi et al. (2019)^38^ and Yousef et al. (2020)^18^ showed that injecting LS brine at intermediate water saturations—after partial HS water injection but before breakthrough—enhances oil recovery by shifting wettability and mobilizing previously trapped oil. These findings directly support our simulation results where LS injection prior to HS breakthrough leads to favorable capillary-to-viscous transitions, increased pore accessibility, and more stable front propagation, as captured by our Regime Index formulation.

Further, the synergistic effect of LS brine and polymer injection has been consistently reported in both early and recent literature. Zahid et al. (2012)^39^ and Wang et al. (2022)^19^ observed that recovery is maximized when polymer is injected after LS brine due to improved sweep efficiency and delayed breakthrough. Their experiments in sandstone cores found recovery improvements of up to 15–20%, attributed to the combined impact of reduced capillary entry pressures and enhanced mobility control—consistent with our simulated increase in oil recovery in injection sequences involving LS-polymer co-injection at intermediate saturations.

Micromodel experiments by Fadaei et al. (2023)^20^ provide further visual confirmation. Their work demonstrated that LS brine injected early minimizes viscous fingering, allowing polymer introduced afterward to form more stable displacement fronts and access bypassed zones. This matches the observed improvements in displacement stability and oil mobilization in our network model, particularly in Sequences 5, 6, and 9.

Using advanced CT-imaging, Zhang et al. (2024)^21^ provided direct evidence of wettability change and phase redistribution during core floods with varying salinity and polymer viscosities. Their results confirmed that LS injection at intermediate saturations improved connectivity and facilitated ganglia mobilization, outcomes which align well with the pressure-gradient-driven displacement shifts and enhanced recovery trends seen in our model.

The importance of injection order was also discussed by Austad et al. (2010)^40^ who found that LS brine injected after HS waterflooding to residual oil saturation (Sor) produced limited incremental recovery. This is attributed to the formation of established HS flow paths that the LS brine cannot disrupt effectively—a limitation also observed in our simulations, where post-Sor LS injection produced marginal gains due to confinement to existing pathways.

Finally, the regime behavior observed in our simulation—characterized by transitions from capillary to viscous dominance—is supported by experimental results and pressure drop trends reported in Sheng (2014)^29^. These results emphasize that injecting LS or polymer too late in the flood cycle limits their effectiveness, a point reinforced by the comparative drop in recovery observed in both literature and our later injection sequences.

Taken together, these experimental validations—ranging from core floods to pore-scale visualization—confirm that the sequence and timing of LS brine and polymer injection are pivotal in determining recovery outcomes. Intermediate saturation injection strategies optimize the balance between wettability alteration and mobility control, and the resulting flow regime transitions are captured both experimentally and within our simulation framework. The alignment of these trends across scales and methodologies affirms the robustness of the conclusions presented in this study.

### Practical implications and economic considerations

The results of this study have important implications for the design and implementation of enhanced oil recovery (EOR) strategies in the petroleum industry. The observed sensitivity of oil recovery to the relative timing of low salinity (LS) brine and polymer injection suggests that injection sequencing should be treated as a key design parameter, rather than a secondary operational detail. By optimizing the timing—particularly by injecting LS brine prior to polymer or co-injecting at intermediate saturations—operators can maximize the synergistic benefits of wettability alteration and improved mobility control. These findings are especially relevant for waterflooded reservoirs transitioning into tertiary recovery phases, where residual oil saturation is high and traditional polymer floods often suffer from poor sweep efficiency.

From an economic perspective, the combination of LS brine and polymer flooding offers a cost-effective alternative to more complex chemical EOR methods such as surfactant-polymer flooding. As noted by Mohammadi and Jerauld (2012)^15^, LS brine can significantly reduce polymer adsorption and degradation, thereby lowering the required polymer dosage and improving the efficiency of the polymer bank. This leads to reduced chemical costs and improved injectivity, particularly in sandstone reservoirs with moderate salinity levels. In addition, by enhancing the effectiveness of polymer flooding, LS preconditioning may reduce the total pore volume injected to reach target recovery levels, translating into shorter project lifecycles and reduced water handling costs.

These qualitative conclusions are supported by field-scale studies with economic metrics. For example, Al-Khafaji et al. (2019)^38^ reported that hybrid LS-polymer injection schemes yielded improved net present value (NPV) and lower cost per incremental barrel compared to polymer-only floods. Similarly, Sheng (2014)^13^ provided a comparative cost analysis of chemical EOR methods, showing that LS-polymer flooding can achieve favorable economics due to reduced polymer consumption and enhanced recovery efficiency. Stavland et al. (2012)^16^ also demonstrated that LS brine can improve polymer injectivity and reduce operational costs associated with chemical loss and water management.

Overall, the findings support the use of LS-polymer hybrid flooding as a scalable and economically attractive EOR strategy, provided that injection timing is properly optimized based on reservoir characteristics. Future field pilots and techno-economic evaluations are encouraged to further validate these simulation-based insights.

## Conclusion and recommendations

This study offers a novel contribution to enhanced oil recovery (EOR) research by systematically investigating the influence of relative timing and sequencing of low salinity (LS) brine and polymer injection on oil recovery using a dynamic pore-scale network model. While previous studies have demonstrated the individual benefits of LS brine and polymer flooding, our results reveal the critical role of their temporal coordination in maximizing recovery. By integrating regime analysis with displacement behavior across multiple capillary networks, we provide mechanistic insight into the interplay between wettability alteration, mobility control, and flow regime evolution.

The study shows that injecting LS brine at intermediate high salinity (HS) saturation—prior to breakthrough—enables more effective wettability alteration and capillary entry pressure reduction, which improves oil recovery compared to LS injection at residual saturation or in secondary mode. Similarly, early or simultaneous polymer injection with LS brine enhances displacement front stability and leads to better areal sweep efficiency. The findings emphasize that the same fluids can yield significantly different recovery outcomes depending on injection timing and sequence.

**Key practical recommendations for field implementation include**:

1. Time LS brine injection to precede breakthrough of HS water, particularly targeting intermediate saturations (e.g., 20–40% PV injected), where wettability alteration can be more uniformly achieved and capillary resistance minimized before residual oil becomes trapped.
2. Co-inject LS brine and polymer in secondary mode to maximize synergy between capillary and viscous mechanisms. This approach yielded the highest simulated recoveries and should be prioritized for pilot testing where both fluids are available early in the flood lifecycle.
3. When LS brine is injected first, avoid injecting it in early secondary recovery or after HS residual saturation is reached, as its effectiveness is reduced once dominant flow paths are established or capillary trapping is complete.
4. Consider injecting polymer before LS brine in reservoirs where high mobility control is required early. This sequence proved to stabilize the displacement front and create favorable conditions for LS brine to access bypassed zones, enhancing overall sweep.
5. Adjust injection strategies based on reservoir flow regimes: LS brine is more impactful in capillary-dominated regimes (e.g., low frontal advance velocity or tight formations), while polymer dominates performance in viscous-dominated regimes (e.g., high injection rates or more permeable zones).
6. In heterogeneous reservoirs or formations with varying wettability, perform pre-screening or limited pilots to evaluate optimal injection timing and order, as small changes in pore structure or mineralogy may shift the optimal sequence.
7. Evaluate polymer concentration and brine salinity interaction to avoid viscosity losses due to dilution. Mixing polymer with LS brine at the surface, rather than injecting LS after polymer, is preferable when viscosity maintenance is critical.

While the analysis was conducted using a 2D pore network model, the trends and mechanisms observed—such as the timing-dependent transition between capillary and viscous regimes, and the sensitivity of recovery to flow stabilization—are expected to hold in three-dimensional systems. Future work should explore 3D extensions and incorporate field-scale reservoir heterogeneity, but the current results already provide a robust framework for guiding the design of low salinity polymer flooding in both lab and field-scale applications^41^.

By incorporating these findings into field development planning, operators can improve the efficiency and predictability of hybrid EOR strategies and reduce uncertainty associated with fluid interaction and reservoir response^42^.

## Data Availability

The datasets generated during and/or analysed during the current study is provided within the manuscript. Any additional data request can be made available by the corresponding author on reasonable request.
